# MALDI-TOF Mass Spectrometry Is a Fast and Reliable Platform for Identification and Ecological Studies of Species from Family *Rhizobiaceae*

**DOI:** 10.1371/journal.pone.0020223

**Published:** 2011-05-31

**Authors:** Laura Ferreira, Fernando Sánchez-Juanes, Paula García-Fraile, Raúl Rivas, Pedro F. Mateos, Eustoquio Martínez-Molina, José Manuel González-Buitrago, Encarna Velázquez

**Affiliations:** 1 Unidad de Investigación, Hospital Universitario de Salamanca, Salamanca, Spain; 2 Departamento de Microbiología y Genética, Universidad de Salamanca, Salamanca, Spain; 3 Departamento de Bioquímica y Biología Molecular, Universidad de Salamanca, Salamanca, Spain; Monash University, Australia

## Abstract

Family *Rhizobiaceae* includes fast growing bacteria currently arranged into three genera, *Rhizobium*, *Ensifer* and *Shinella*, that contain pathogenic, symbiotic and saprophytic species. The identification of these species is not possible on the basis of physiological or biochemical traits and should be based on sequencing of several genes. Therefore alternative methods are necessary for rapid and reliable identification of members from family *Rhizobiaceae*. In this work we evaluated the suitability of Matrix-Assisted Laser Desorption Ionization-Time-of-Flight Mass Spectrometry (MALDI-TOF MS) for this purpose. Firstly, we evaluated the capability of this methodology to differentiate among species of family *Rhizobiaceae* including those closely related and then we extended the database of MALDI Biotyper 2.0 including the type strains of 56 species from genera *Rhizobium, Ensifer* and *Shinella*. Secondly, we evaluated the identification potential of this methodology by using several strains isolated from different sources previously identified on the basis of their *rrs*, *recA* and *atpD* gene sequences. The 100% of these strains were correctly identified showing that MALDI-TOF MS is an excellent tool for identification of fast growing rhizobia applicable to large populations of isolates in ecological and taxonomic studies.

## Introduction

The family *Rhizobiaceae* currently contains fast growing species of bacteria that may be saprophytic or able to establish beneficial or deleterious plant interactions. These species are currently arranged into three genera, *Rhizobium*, *Ensifer* and *Shinella* [1], [2]. The former genera *Agrobacterium* and *Allorhizobium* are now included in genus *Rhizobium* [3] and *Sinorhizobium* is currently named *Ensifer* [4]. The identification of members of the family *Rhizobiaceae* is necessarily based on gene sequencing since there is not phenotypic information that allows the differentiation and identification of rhizobial species [3]. Therefore, although gene sequencing is the most reliable method for identification of rhizobia, it is still a tedious and time-consuming method to be applied to wide populations and therefore alternative methods are necessary for reliable identification of these bacteria shortening the time needed to achieve this process.

Matrix-assisted laser desorption ionization–time-of-flight mass spectrometry (MALDI-TOF MS) has been suggested as a fast and reliable method for bacterial identification, based on the characteristic protein profiles for each microorganism. Using this technology it has been estimated that up to 99% of strains tested are correctly identified when comparing with commercial phenotypic identification panels or *rrs* gene sequencing [5]–[8]. However MALDI-TOF MS has been basically applied to the identification of clinical isolates [9]–[16] so most of the species currently included on available databases are those of clinical interest. For example in the case of family *Rhizobiaceae* only the type strains of three species, *Rhizobium tropici*, *Rhizobium radiobacter* and *Rhizobium rubi*, and eight pathogenic non-type strains of *R. radiobacter*, *R. rhizogenes* and *Agrobacterium tumefaciens* (currently *R. radiobacter*) are included in Biotyper 2.0 database (Bruker Daltonics) used in this study.

Therefore the objectives of this work were: (i) the evaluation of MALDI-TOF MS technology for species differentiation within family *Rhizobiaceae*, (ii) the construction of a database that includes the type strains of currently accepted species within family *Rhizobiaceae* and (iii) the validation of the MALDI-TOF MS technology to identify rhizobial strains isolated from nodules and tumours previously identified by gene sequencing.

## Materials and Methods

### Bacterial strains and culture conditions

To build a reference database for MALDI-TOF MS-based rhizobial species identification, the type strains of 56 species belonging to the family *Rhizobiaceae* were used (Table 1). In addition 35 strains isolated from legume nodules or plant tumours previously characterized by gene sequencing were used to validate MALDI-TOF MS as an identification tool for this group of bacteria (Table 2).

**Table 1:** Type strains of family *Rhizobiaceae* included in the extended database for MALDI-TOF MS- based species identification.

Species	Strains included in database	Source of isolation	References
*Ensifer adhaerens*	LMG 20216^T^	soil	[50]
*Ensifer americanum*	DSM 15007^T^	*Acacia* nodules	[36], [51]
*Ensifer arboris*	LMG 14919^T^	*Prosopis chilensis* nodules	[36], [52]
*Ensifer fredii*	USDA 205^T^, LMG 6217^T^	*Glycine max* nodules	[36], [53], [54]
*Ensifer garamanticus*	LMG 24692^T^	*Agryrolobium uniflorum* nodules	[55]
*Ensifer kostiense*	LMG 19227^T^	*Acacia senegal* nodules	[36], [52]
*Ensifer kummerowiae*	CCBAU 71714^T^	*Kummerowia stipulacea* nodules	[36], [56]
*Ensifer medicae*	USDA 1037^T^	*Medicago truncatula* nodules	[36], [57]
*Ensifer meliloti*	ATCC 9930^T^	*Medicago sativa* nodules	[36], [58]
*Ensifer morelense*	Lc04^T^	*Leucaena leucocephala* nodules	[36], [59]
*Ensifer numidicus*	LMG 24690^T^	*Agryrolobium uniflorum* nodules	[55]
*Ensifer saheli*	LMG 7837^T^	*Sesbania cannabina* nodules	[36], [58]
*Ensifer terangae*	LMG 7834^T^	*Acacia laeta* nodules	[36], [58]
*Rhizobium aggregatum*	DSM 1111^T^	Surface lake water	[30], [60]
*Rhizobium alamii*	LMG 24466^T^	Plant rhizosphere	[61]
*Rhizobium alkalisoli*	DSM 21826^T^	*Caragana microphylla* nodules	[62]
*Rhizobium borbori*	LMG 23925^T^	Activated sludge	[63]
*Rhizobium cellulosilyticum*	ALA10B2^T^	*Populus alba* sawdust	[64]
*Rhizobium daejeonense*	DSM 17795^T^	Cyanide treatment bioreactor	[65]
*Rhizobium etli*	CFN42^T^	*Phaseolus vulgaris* nodules	[32]
*Rhizobium fabae*	LMG 23997^T^	*Vicia faba* nodules	[66]
*Rhizobium galegae*	HAMBI 540^T^	*Galega orientalis* nodules	[67]
*Rhizobium gallicum*	R602sp^T^	*Phaseolus vulgaris* nodules	[45]
*Rhizobium giardinii*	H152^T^	*Phaseolus vulgaris* nodules	[45]
*Rhizobium hainanense*	I66^T^	*Desmodium sinuatum* nodules	[68]
*Rhizobium huautlense*	S02^T^	*Sesbania herbacea* nodules	[69]
*Rhizobium indigoferae*	CCBAU 71042^T^	*Indigofera amblyantha* nodules	[56]
*Rhizobium larrymoorei*	LMG 21410^T^	*Ficus benjamina* aerial tumours	[3], [70], [71]
*Rhizobium leguminosarum*	USDA 2370^T^	*Pisum sativum* nodules	[29]
*Rhizobium loessense*	CCBAU 7190B^T^, LMG23187^T^	*Astragalus complanatus* nodules	[72]
*Rhizobium lusitanum*	p1-7^T^	*Phaseolus vulgaris* nodules	[48]
*Rhizobium mesosinicum*	LMG 24135^T^	*Albizia julibrissin*	[73]
*Rhizobium miluonense*	LMG 24208^T^	*Lespedeza chinensis* nodules	[74]
*Rhizobium mongolense*	USDA 1844^T^	*Medicago ruthenica* nodules	[75]
*Rhizobium multihospitium*	LMG 23946^T^	*Halimodendron halodendron* nodules	[76]
*Rhizobium oryzae*	LMG 24253^T^	Wild rice surface sterilized roots	[77]
*Rhizobium phaseoli*	ATCC 14482^T^	*Phaseolus vulgaris* nodules	[29], [32]
*Rhizobium pisi*	DSM 30132^T^	*Pisum sativum* nodules	[29]
*Rhizobium radiobacter* (formerly *Agrobacterium radiobacter*)	ATCC 19358^T^, NCBI 9042^T^, CIP 104325^T^	Soil	[3], [28]
*Rhizobium rhizogenes*	ATCC 11325^T^	Hairy roots	[3], [28]
*Rhizobium rosettiformans*	CCM 7583^T^	hexachlorocyclohexane (HCH) dump site	[30]
*Rhizobium rubi*	ATCC 13335^T^, DSM 6772^T^	*Rubus* tumours	[3], [78]
*Rhizobium selenitireducens*	LMG 24075^T^	Cyanide treatment bioreactor	[79]
*Rhizobium sullae*	IS 123^T^	*Hedysarum coronarium* nodules	[80]
*Rhizobium tibeticum*	DSM 21102^T^	*Trigonella archiducis-nicolai*	[81]
*Rhizobium tropici*	CIAT 899^T^	*Leucaena leucocephala* nodules	[82]
*Rhizobium tubonense*	LMG 25225^T^	*Oxytropis glabra* nodules	[83]
*Rhizobium undicola*	LMG 11875^T^	*Neptunia natans* nodules	[3], [84]
*Rhizobium vitis*	CECT 4799^T^	*Vitis vinifera* tumours	[3], [85]
*Rhizobium vignae*	LMG 25447^T^	*Vigna* nodules	[86]
*Rhizobium yanglingense*	CCBAU 71623^T^	*Gueldenstaedtia* nodules	[87]
*Shinella granuli*	DSM 18401^T^	Upflow anaerobic sludge blanket reactor	[88]
*Shinella fusca*	LMG 24714^T^	Domestic waste compost	[89]
*Shinella kummerowiae*	LMG 24136^T^	*Kummerowia stipulacea* nodules	[2]
*Shinella yambaruensis*	DSM 18801^T^	Soil	[90]
*Shinella zoogloeoides*	DSM 287^T^	Activated sludge	[88], [91]

**Table 2:** MALDI BioTyper identification results for family *Rhizobiaceae* strains.

	Source of isolation	Reference	Organism (best match)	score values*
**A. Strains from different collections**				
*Ensifer fredii* USDA 205^T^	*Glycine* nodules	[53]	*Ensifer fredii* LMG 6217^T^	2.585
*Rhizobium loessense* LMG 23187^T^	*Astragalus* nodules	[72]	*Rhizobium loessense* CCBAU 7190B^T^	2.335
*Rhizobium tropici* CIAT 899^T^	*Phaseolus* nodules	[82]	*Rhizobium tropici* DSM 11418^T^	2.582
*Rhizobium radiobacter* ATCC 19358^T^ (NCBI 9042^T^, CIP 104325^T^)	soil	[28]	*Rhizobium radiobacter* DSM 30147^T^	2.566 (2.524, 2.488)
*Rhizobium rubi* ATCC 13335^T^	*Rubus* tumours	[28]	*Rhizobium rubi* DSM 6772^T^	2.505
**B. Species correctly reclassified in other species**				
*Agrobacterium tumefaciens* ATCC 23308 (NCBI 13307, CIP 67.1), former type strains	*Malus* tumours	[28]	*Rhizobium radiobacter* DSM 30147^T^ (strain included in Biotyper 2.0 database)	2.522 (2.408, 2.405)
*Ensifer xinjiangense* LMG 17930 (CECT 4657), former type strains	*Glycine* nodules	[92]	*Ensifer fredii* LMG 6217^T^	2.413 (2.151)
*Rhizobium trifolii* ATCC 14480, former type strain	*Trifolium* nodules	[29]	*Rhizobium leguminosarum* USDA 2370^T^	2.128
**C. Species erroneously included in other species**				
*Ensifer morelense* Lc04^T^	*Leucaena* nodules	[36], [59]	*Ensifer adhaerens* LMG 20216^T^	1.245
*Rhizobium phaseoli* ATCC 14482^T^	*Phaseolus* nodules	[29], [32]	*Rhizobium etli* CFN42^T^	1.991
*Rhizobium pisi* DSM 30132^T^	*Pisum* nodules	[29]	*Rhizobium leguminosarum* USDA 2370^T^	1.782
**D. Species with problems in their identity**				
*Ensifer kummerowiae* CCBAU 71714^T^	*Kummerowia* nodules	[56]	*Ensifer meliloti* ATCC 9930^T^	2.261
*Rhizobium fabae* LMG 23997^T^	*Vicia* nodules	[66]	*Rhizobium pisi* DSM 30132^T^	2.258
*Rhizobium indigoferae* CCBAU 71042^T^	*Indigofera* nodules	[56]	*Rhizobium leguminosarum* USDA 2370^T^	2.219
*Rhizobium loessense* CCBAU 7190B^T^ (LMG23187^T^)	*Astragalus* nodules	[72]	*Rhizobium gallicum* R602sp^T^	2.283 (2.354)
*Rhizobium mongolense* USDA 1844^T^	*Medicago* nodules	[75]	*Rhizobium gallicum* R602sp^T^	2.506
*Rhizobium yanglingense* CCBAU 1623^T^	*Gueldenstaedtia* nodules	[87]	*Rhizobium gallicum* R602sp^T^	2.314
**F. Strains included in already described species**				
RTM17	*Trigonella* nodules	[49]	*Ensifer meliloti* ATCC 9930^T^	2.140
GVPV12	*Phaseolus* nodules	[44]	*Ensifer meliloti* ATCC 9930^T^	2.145
RPA13	*Prosopis* nodules	[43]	*Ensifer meliloti* ATCC 9930^T^	2.241
RMP01	*Melilotus* nodules	[49]	*Ensifer medicae* USDA 1037^T^	2.252
RMP05	*Melilotus* nodules	[49]	*Ensifer medicae* USDA 1037^T^	2.114
RPA08	*Prosopis* nodules	[43]	*Ensifer medicae* USDA 1037^T^	2.092
RPA11	*Prosopis* nodules	[43]	*Ensifer medicae* USDA 1037^T^	2.177
RPA20	*Prosopis* nodules	[43]	*Ensifer medicae* USDA 1037^T^	2.211
FL27	*Phaseolus* nodules	[45]	*Rhizobium gallicum* R602sp^T^	2.405
PhD12	*Phaseolus* nodules	[45]	*Rhizobium gallicum* R602sp^T^	2.399
RPA02	*Prosopis* nodules	[43]	*Rhizobium giardinii* H152^T^	2.432
RPA12	*Prosopis* nodules	[43]	*Rhizobium giardinii* H152^T^	2.425
RPVF18	*Phaseolus* nodules	[38]	*Rhizobium leguminosarum* USDA 2370^T^	2.017
RVS11	*Vicia* nodules	[39]	*Rhizobium leguminosarum* USDA 2370^T^	2.152
ATCC 14480	*Trifolium* nodules	[29]	*Rhizobium leguminosarum* USDA 2370^T^	2.128
P3-13	*Phaseolus* nodules	[48]	*Rhizobium lusitanum* P1-7^T^	2.314
USDA 1929	*Medicago* nodules	[75]	*Rhizobium mongolense* USDA1844^T^	2.474
ATCC 13332	no data	[93]	*Rhizobium radiobacter* DSM 30147^T^	2.644
163C	*Prunus* tumours	[47]	*Rhizobium rhizogenes* ATCC 11325^T^	2.195
IAM 13571	no data	[48]	*Rhizobium rhizogenes* ATCC 11325^T^	2.267
K84	soil	[46]	*Rhizobium rhizogenes* ATCC 11325^T^	2.185
Br859	*Leucaena* nodules	[82]	*Rhizobium tropici* CIAT 899^T^	2.613
**G. Strains do not belonging to described species**				
Br816	*Leucaena* nodules	[34], [35]	*Ensifer americanum*	1.775
RPVR32	*Phaseolus* nodules	[38]	*Rhizobium leguminosarum* USDA 2370^T^	1.066
CVIII4	*Vicia* nodules	[39]	*Rhizobium leguminosarum* USDA 2370^T^	1.288
RTP05	*Trifolium* nodules	[94]	*Rhizobium leguminosarum* USDA 2370^T^	1.701
CFN299	*Leucaena* nodules	[82]	*Rhizobium tropici* CIAT 899^T^	1.191
C58	*Prunus* tumour	[95]	*Rhizobium radiobacter* DSM 30147^T^	1.956

*score value ≥2 indicates species identification; 1.7<score value<2 indicates genus identification, score value <1.7 indicates no identification.

In order to establish the more adequate medium and growth conditions for fast-growing rhizobia analysis, selected strains were cultivated on TY [17] and YMA plates [18] and incubated at 28°C for 24 and 48 hours.

### Sample preparation for MALDI-TOF MS

Cells of a whole colony were transferred from the plate to a 1.5 ml tube (Eppendorf, Germany) with a pipette tip and mixed thoroughly in 300 µl of water to resuspend the bacterial cells. Then, 900 µl of absolute ethanol was added and the mixture was centrifuged at 15,500 *g* for 2 min, and the supernatant was discarded. The pellet was air-dried at room temperature for 1 hour. Subsequently, 50 µl of formic acid (70% v/v) was added to the pellet and mixed thoroughly before the addition of 50 µl acetonitrile to the mixture. The mixture was centrifuged again at 15,500 *g* for 2 min. One microliter of the supernatant was placed onto a spot of the steel target and air-dried at room temperature. Each sample was overlaid with 1 µl of matrix solution and air-dried.

### MALDI-TOF MS

Measurements were performed on an Autoflex III MALDI-TOF/TOF mass spectrometer (Bruker Daltonics, Leipzig, Germany) equipped with a 200-Hz smartbeam laser. Spectra were recorded in the linear, positive mode at a laser frequency of 200 Hz within a mass range from 2,000 to 20,000 Da. The IS1 voltage was 20 kV, the IS2 voltage was maintained at 18.6 kV, the lens voltage was 6 kV, and the extraction delay time was 40 ns.

For each spectrum, 500 laser shots were collected and analyzed (10×50 laser shots from different positions of the target spot). The spectra were calibrated externally using the standard calibrant mixture (*Escherichia coli* extracts including the additional proteins RNase A and myoglobin, Bruker Daltonics). Calibration masses were as follows: RL36, 4364.3 Da; RS22, 5095.8 Da; RL34, 5380.4 Da; RL33meth, 6254.4 Da; RL32, 6315 Da; RL29, 7273.5 Da; RS19, 10299.1 Da; RNase A, 13682.2 Da; myoglobin, 16952.5 Da.

### Spectrum generation and data analysis

For automated data analysis, raw spectra were processed using the MALDI Biotyper 2.0 software (Bruker Daltonics, Leipzig, Germany) at default settings. The software performs normalization, smoothing, baseline subtraction, and peak picking, creating a list of the most significant peaks of the spectrum (*m/z* values with a given intensity, with the threshold set to a minimum of 1% of the highest peak and a maximum of 100 peaks). To identify unknown bacteria, each peak list generated was matched directly against reference libraries (3,476 species) using the integrated patterns matching algorithm of the Biotyper 2.0 software (Bruker Daltonics, GmbH, Germany). The unknown spectra were compared with a library of reference spectra based on a pattern recognition algorithm using peak position, peak intensity distributions and peak frequencies. Once a spectrum has been generated and captured by the software, the whole identification process was performed automatically, without any user intervention. MALDI-TOF MS identifications were classified using modified score values proposed by the manufacturer: a score value ≥2 indicated species identification; a score value between 1.7 and 1.9 indicated genus identification, and a score value <1.7 indicated no identification.

For reference library construction, 36 independent spectra were recorded for each bacterial isolate (three independent measurements at twelve different spots each). Manual/visual estimation of the mass spectra was performed using Flex Analysis 3.0 (Bruker Daltonics GmbH, Germany) performing smoothing and baseline substraction. Checking existence of flatlines, outliers or single spectra with remarkable peaks differing from the other spectra was done, taking into account that mass deviation within the spectra set shall not be more than 500 ppm. Finally, 20 spectra were selected, removing questionable spectra from the collection. To create peak lists of the spectra, the BioTyper software was used as described above. The 20 independent peak lists of a strain were used for automated “main spectrum” generation with default settings of the BioTyper software. Thereby, for each library entry a reference peak list (main spectrum) which contains information about average masses, average intensities, and relative abundances in the 20 measurements for all characteristic peaks of a given strain was created, so a main spectrum displayed the most reproducible peaks typical for a certain bacterial strain.

Cluster analysis was performed based on comparison of strain-specific main spectra created as described above. The dendrogram was constructed by the statistical toolbox of Matlab 7.1 (MathWorks Inc., USA) integrated in the MALDI Biotyper 2.0 software. The parameter settings were: ‘Distance Measure = Euclidian’ and ‘Linkage = complete’. The linkage function is normalized according to the distance between 0 (perfect match) and 1000 (no match).

### Phylogenetic analyses

The results of MALDI-TOF MS analysis were compared with those obtained after *rrs*, *recA*, *atpD* and *nodC* gene sequence analyses. In this work we obtained some sequences of these genes that are absent in databases according to Rivas *et al.* [19] for *rrs* gene, Gaunt *et al.* [20] for *recA* and *atpD* genes and Laguerre *et al.* [21] for *nodC* gene. The sequences were aligned using the Clustal W software [22]. The distances were calculated according to Kimura's two-parameter model [23]. Phylogenetic trees were inferred using the neighbour-joining method [24] and the MEGA 4.0 package [25].

## Results and Discussion

### Database setting

In Biotyper 2.0 database only three species of genus *Rhizobium* are included and none of genus *Ensifer* or *Shinella*. Therefore a database extension in order to include the species currently described in these genera is necessary before applying MALDI-TOF MS to the identification of rhizobial isolates.

Owing to the fact that in Biotyper 2.0 database the type strains of three species of genus *Rhizobium* are already included, *R. tropici* DSM 11418^T^, *R. rubi* DSM 6772^T^ and *R. radiobacter* DSM 30147^T^, we verified the reproducibility of MALDI-TOF MS using the type strains of these species that were cultivated in two different media (YMA and TY) and incubated at 24 and 48 h.

The results obtained showed that the analysed strains matched with high score values (higher than 2.5) with each corresponding type strain already present in Biotyper 2.0 database when TY medium and 24 h incubation were used (Table 3). Lower score values were found with YMA medium incubated at 24 h and only *R. rubi* ATCC 13335^T^ and *R. radiobacter* ATCC 19358^T^ were correctly identified with score values higher than 2 (Table 3). This was probably due to the production of higher amounts of exopolysaccharide in YMA medium which makes the sample preparation difficult. After an incubation time of 48 h the score values were lower when both TY and YMA media were used and only *R. rubi* ATCC 13335^T^ and *R. radiobacter* ATCC 19358^T^ were correctly identified using YMA medium. Therefore best results for rhizobial species were obtained with TY medium and 24 h incubation, in spite of previous studies that have demonstrated high reproducibility of MALDI-TOF MS analysis in different culture media and growth phases [14], [26], [27].

**Table 3:** Comparison of identification results by MALDI-TOF MS with different conditions.

Media and incubation times (extraction method)				
Strain	Incubated in YMA during 24 h (best match)	Score value*	Incubated in TY during 24 h (best match)	Score value*
*Rhizobium radiobacter* ATCC 19358^T^	*Rhizobium radiobacter* DSM 30147^T^	2.388	*Rhizobium radiobacter* DSM 30147^T^	2.566
*Rhizobium tropici* CIAT 899^T^	*Rhizobium tropici* DSM 11418^T^	1.897	*Rhizobium tropici* DSM 11418^T^	2.582
*Rhizobium rubi* ATCC 13335^T^	*Rhizobium rubi* DSM 6772^T^	2.500	*Rhizobium rubi* DSM 6772^T^	2.505

Strain	Incubated in YMA during 48 h (best match)	Score value*	Incubated in TY during 48 h (best match)	Score value*
*Rhizobium radiobacter* ATCC 19358^T^	*Rhizobium radiobacter* DSM 30147^T^	2.077	*Rhizobium radiobacter* DSM 30147^T^	1.389
*Rhizobium tropici* CIAT 899^T^	*Rhizobium tropici* DSM 11418^T^	1.399	*Rhizobium tropici* DSM 11418^T^	1.547
*Rhizobium rubi* ATCC 13335^T^	*Rhizobium rubi* DSM 6772^T^	2.333	*Rhizobium rubi* DSM 6772^T^	1.522

*score value ≥2 indicates species identification; 1.7<score value<2 indicates genus identification, score value <1.7 indicates no identification.

Before the extension of Biotyper 2.0 database we also checked the suitability of MALDI-TOF MS system to differentiate the spectra of representative species from the three genera currently accepted in Family *Rhizobiaceae*.

**Figure 1:**
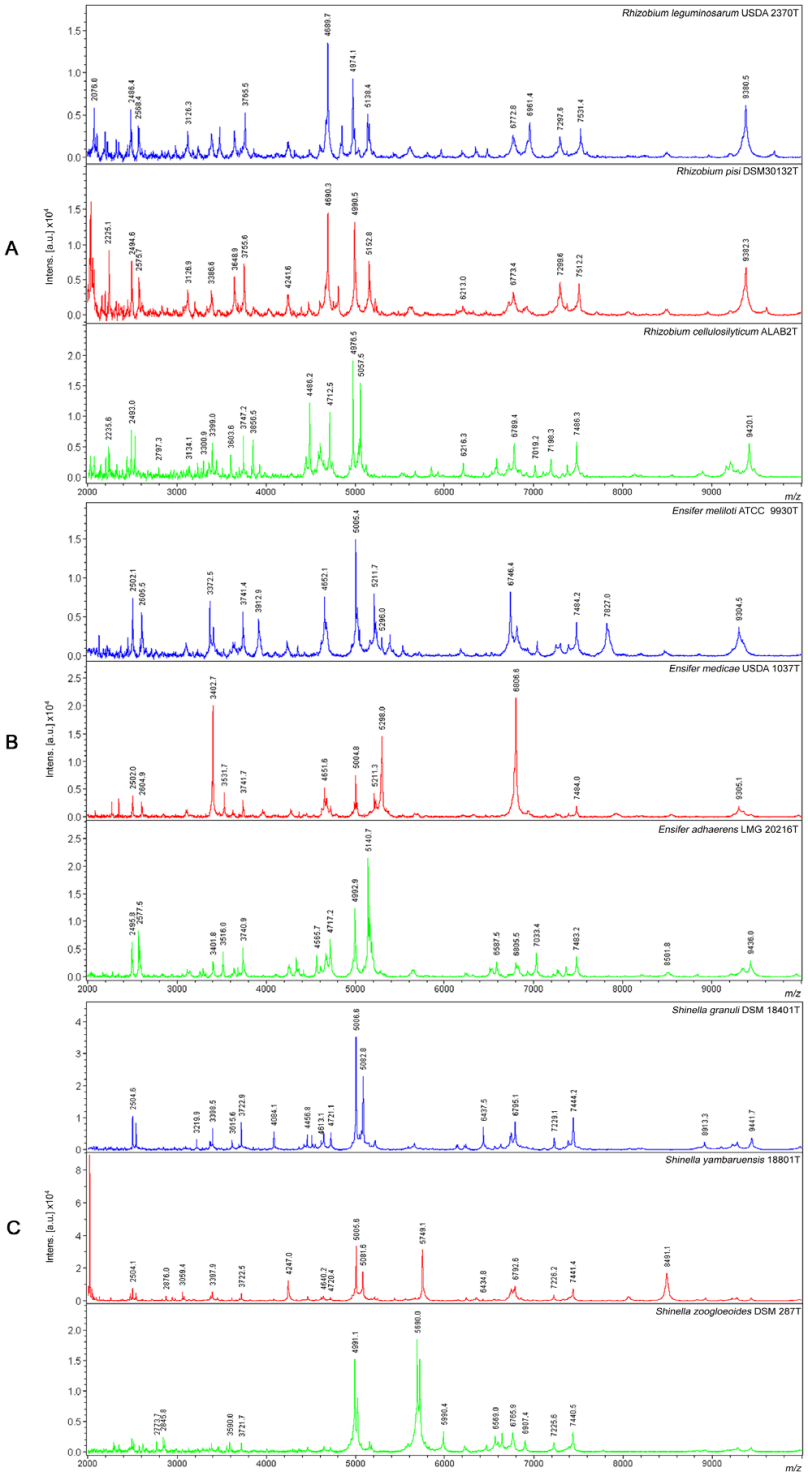
MALDI-TOF MS spectra of whole-cell extracts obtained from the type strains of two close and one divergent species from each genus analysed in this study: (A) *Rhizobium*, (B) *Ensifer* and (C) *Shinella*.

Firstly we compared the spectra of the type strains from the type species of the three genera currently included in family *Rhizobiaceae*. The results obtained showed that the spectra of *Rhizobium leguminosarum* USDA 2370^T^, *Ensifer adhaerens* LMG 20216^T^ and *Shinella granuli* DSM18401^T^ were clearly distinguishable since there were not common peaks among their spectra (figure 1).

Subsequently, we analyzed the spectra of two phylogenetically close and one phylogenetically divergent species from each genus. We selected from genus *Rhizobium* the close species *R. leguminosarum* (type species of genus *Rhizobium*) and *R. pisi* as well as the species *R. cellulosilyticum*, phylogenetically distant from them. From genus *Ensifer* we chose the close species *E. meliloti* and *E. medicae* and the species *E. adhaerens*, which is the type species of genus *Ensifer* and it is phylogenetically distant from the other two species. Finally, from genus *Shinella* we chose the close species *S. granuli*, type species of genus *Shinella*, and *S. kummerowiae* and the phylogenetically distant *S. fusca*.

All these spectra were quite different with almost any common peaks among those of species belonging to different genera as we previously observed for the type species of each genus. Within the same genus the spectra of close species were more similar than those from divergent species. For example, considering the mass tolerance ±2 m/z for each peak as we have previously described [13], in genus *Rhizobium*, *R. leguminosarum* USDA 2370^T^ and *R. pisi* DSM 30132^T^ shared peaks at 3126, 4689, 6773, 7298 and 9380 Da that are not in *R. cellulosilyticum* ALAB2^T^ (figure 1A). In genus *Ensifer*, peaks at 2502, 2605, 4652, 5005, 5211 and 9304 Da were present in *E. meliloti* ATCC 9930^T^ and *E. medicae* USDA 1037^T^ and not in *E. adherens* LMG 20216^T^, although there are two common peaks in the three species compared (3741 and 7484 Da). (figure 1B). In genus *Shinella*, we found peaks at 5006, 4613 and 2504 Da in *S. granuli* and *S. kummerowiae* that were not present in *S. fusca* and, although *S. fusca* shared few peaks with *S. granuli* (3615, 3723, 7229, 7444 Da), this phylogenetically distant species had many specific peaks that were not in the other two species (2070, 2237, 2326, 2497, 3254, 5096, 6351, 6507 Da) (figure 1C).

These results showed that the spectra of both phylogenetically close and distant species from the same genus, as well as those of species of different genera within family *Rhizobiaceae* can be differentiated by MALDI-TOF MS. Therefore we extended the database MALDI BioTyper 2.0 with 56 type strains of species from genera *Rhizobium*, *Ensifer* and *Shinella* belonging to Family *Rhizobiaceae* (Table 1).

### Comparison between MALDI-TOF MS and phylogenetic analyses

**Figure 2:**
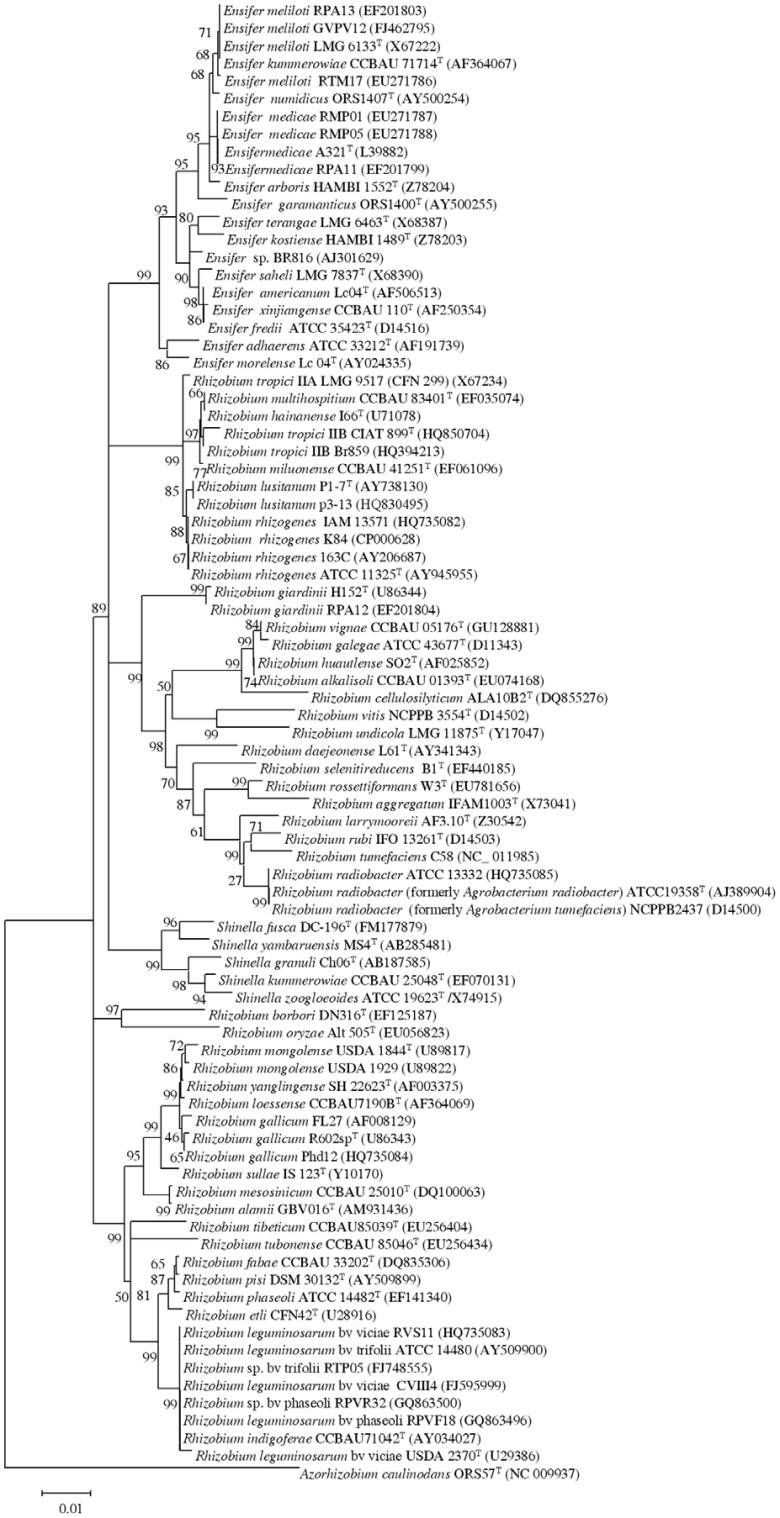
Neighbour-joining phylogenetic rooted tree based on 16S rRNA sequences (about 1475 nt) showing the taxonomic location of the species included in this study. Bootstrap values calculated for 1000 replications are indicated. Bar, 1 nt substitution per 100 nt. Accesion numbers from Genbank are given in brackets.

**Figure 3:**
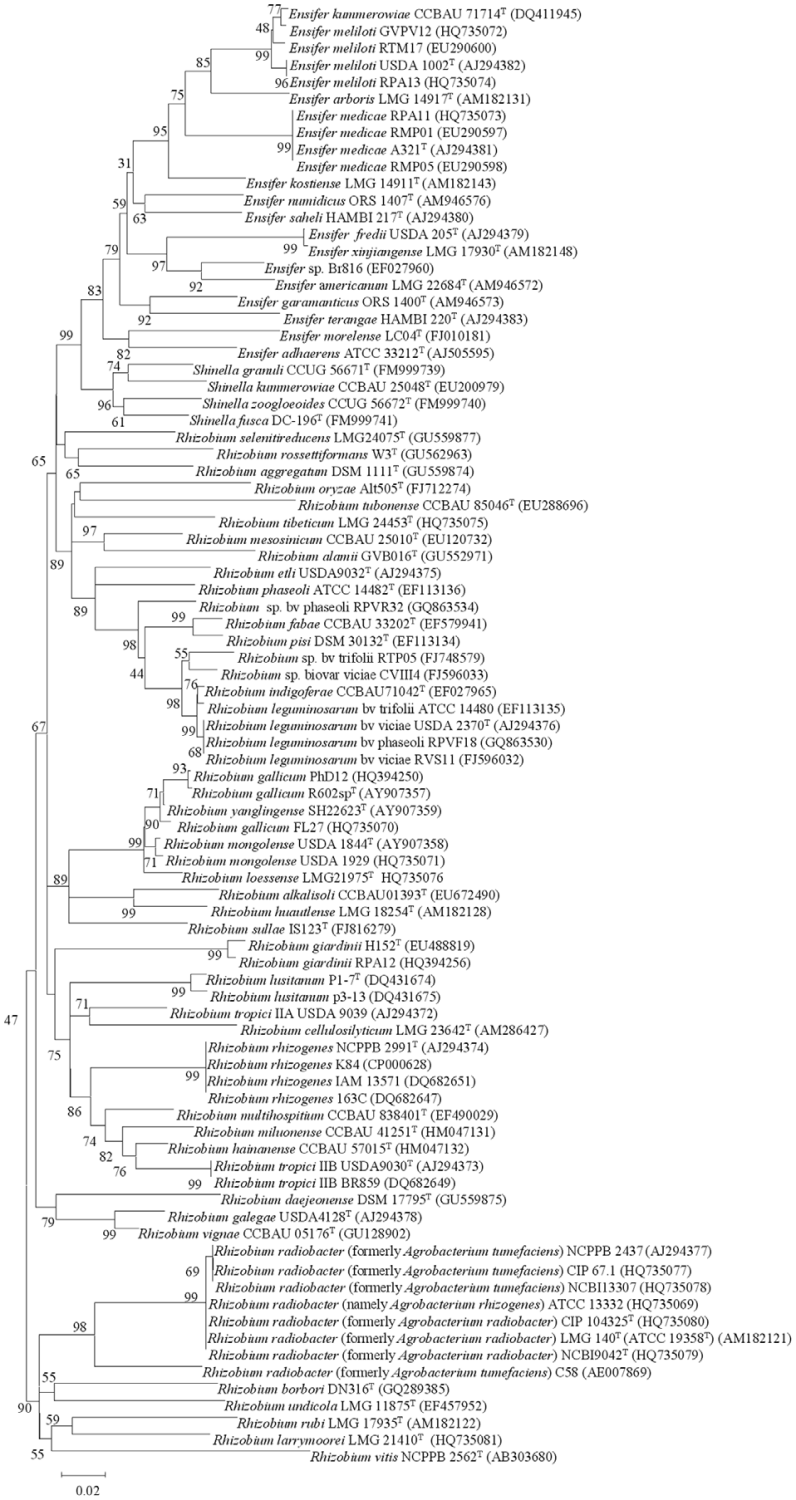
Neighbour-joining phylogenetic tree based on *recA* gene sequences (about 520 nt) showing the position of species included in this study. Bootstrap values calculated for 1000 replications are indicated. Bar, 2 nt substitution per 100 nt. Accesion numbers from Genbank are given in brackets.

**Figure 4:**
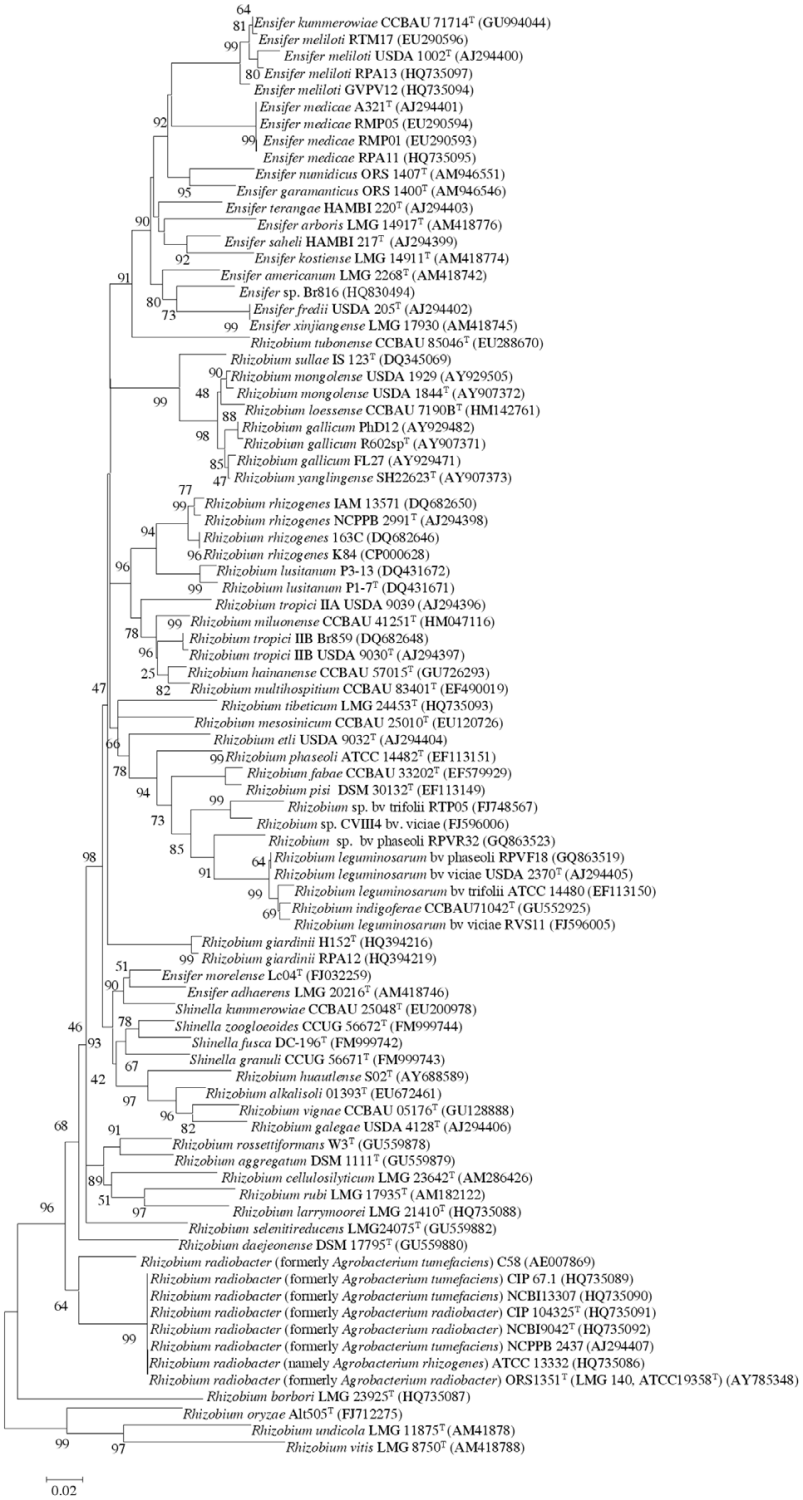
Neighbour-joining phylogenetic tree based on *atpD* gene sequences (about 500 nt) showing the position of species included in this study. Bootstrap values calculated for 1000 replications are indicated. Bar, 2 nt substitution per 100 nt. Accesion numbers from Genbank are given in brackets.

**Figure 5:**
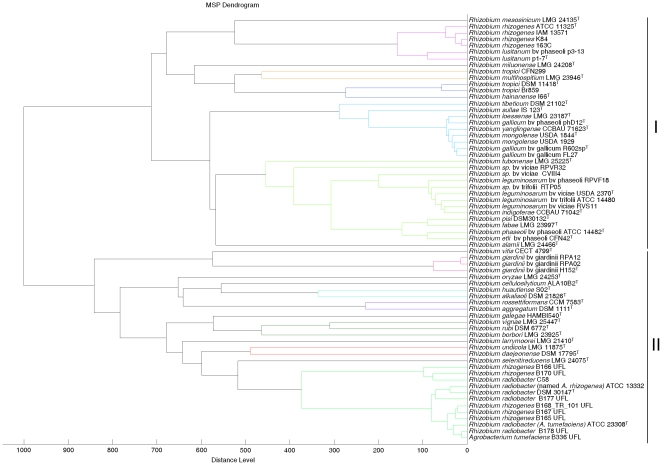
Cluster analysis of MALDI-TOF MS spectra of different species and strains from the genus *Rhizobium*. Distance is displayed in relative units.

To compare the data obtained by MALDI-TOF MS analysis with those based on gene sequence analysis (figures 2, 3 and 4), a cluster analysis was performed based on a correlation matrix using the integrated tools of the MALDI Biotyper 2.0 software package. Figure 5 showed that the genus *Rhizobium* was divided into several clusters whose distribution basically coincided with that observed after *rrs*, *recA* and *atpD* gene analyses. The results evidenced that some reclassifications performed within genus *Rhizobium* are correct as occurs in the case of the former species *Agrobacterium tumefaciens* reclassified into *A. radiobacter* [28]. MALDI-TOF MS results confirmed that they are the same species since their type strains held in different collections matched with score values higher than 2 (Table 2A and 2B). These results are congruent with those obtained from *recA* and *atpD* gene analyses since these strains presented nearly identical sequences (figures 3 and 4). After reclassification of the complete genus *Agrobacterium* into *Rhizobium*, the current valid name for these species is *Rhizobium radiobacter* [3].

MALDI-TOF MS analysis also confirmed the *R. trifolii* ATCC 14480 reclassification into *R. leguminosarum* [29], since the strain ATCC 14480 matched with *R. leguminosarum* USDA 2370^T^ with a score value higher than 2 (Table 2B), and *Blastobacter aggregatus* DSM 1111^T^ into *R. aggregatum* [30] since strain DSM 1111^T^ clustered with species of genus *Rhizobium* (Figure 5).

On the contrary, some species of genus *Rhizobium* were erroneously reclassified. For example, *R. phaseoli* type strain was reclassified into *R. leguminosarum* [31]. Later the biovar phaseoli type I of this species was reclassified into *R. etli* [32], so it was not clear the location of the *R. phaseoli* type strain. A revision based on *rrs*, *recA* and *atpD* analysis showed that *R. phaseoli* is a valid species distinguishable from both *R. leguminosarum* and *R. etli* [29]. The results of the MALDI-TOF MS confirmed these results since *R. phaseoli* ATCC 14482^T^ matched with *R. etli* CFN42^T^ with score values lower than 2 (Table 2C).

Moreover, the MALDI-TOF MS cluster analysis showed, in agreement with *rrs*, *recA* and *atpD* gene analyses, that some current *Rhizobium* species are indistinguishable (figures 2, 3, 4 and 5). For example, the type strains of *R. mongolense*, *R. loessense* and *R. yanglingense* matched with *R. gallicum* R602sp^T^ with score values higher than 2 (Table 2D). In addition, *R. indigoferae* CCBAU 71042^T^ matched with *R. leguminosarum* USDA 2370^T^ with a score value of 2.219 and *R. fabae* LMG 23997^T^ matched with *R. pisi* DSM 30132^T^ with a score value of 2.258 (Table 2D). Therefore the taxonomic status of all these species should be revised according to the current rules of bacterial taxonomy.

**Figure 6:**
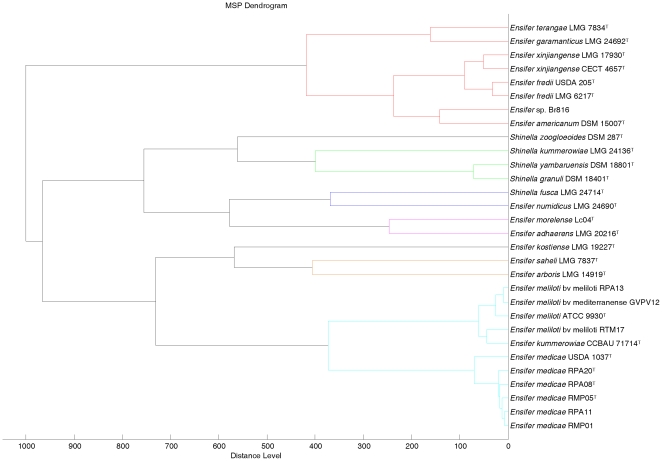
Cluster analysis of MALDI-TOF MS spectra of different species and strains from the genera *Ensifer* and *Shinella*. Distance is displayed in relative units.

The genera *Shinella* and *Ensifer* MALDI-TOF cluster analysis was performed together (figure 6) since they are closely related on the basis of *recA* and *atpD* gene analyses (see figures 3 and 4). This closeness was confirmed after MALDI-TOF cluster analysis although the distribution of *Shinella* species was slightly different (figure 6). The species *S. yambaruensis* was the closest related species to *S. granuli* on the basis of MALDI-TOF MS analysis, whereas these two species were distant according to their *rrs* gene sequences (figure 2). However, *S. yambaruensis* DSM 18801^T^ matched with *S. granuli* DSM 18401^T^ with a score value lower than 2 corresponding to different species from the same genus.

The distribution of species in the genus *Ensifer* was coherent with those found after *rrs* analysis with *E. medicae* and *E. meliloti* forming the same group, *E. morelense* close to *E. adhaerens* and *E. americanum* (a not yet validated species) close to *E. fredii* (figure 6).

In the genus *Ensifer* also MALDI-TOF MS analysis confirmed some reclassifications as that of species *E. xinjiangense* into *E. fredii* [33] since the former type strains *E. xinjiangense* LMG 17930 and CECT 4657 matched with *E. fredii* LMG 6217^T^ with score values of 2.413 and 2.151, respectively (Table 2B). Also was confirmed the reclassification of the strain *Rhizobium* sp. Br816 as *Ensifer* sp. [34], [35] since it clustered with *E. americanum* (figure 6). However, in agreement with *rrs*, *recA* and *atpD* gene analyses (figures 2, 3 and 4), strain Br816 does not belong to this species since it matched with *E. americanum* DSM 15007^T^ with score values lower than 2 (Table 2G).

However, other reclassifications were not correct as occurs with *E. morelense* reclassified into *E. adhaerens* [36] since *E. morelense* Lc04^T^ matched with *E. adhaerens* LMG 20216^T^ with a score value of only 1.245 (Table 2C) in agreement with *rrs*, *recA* and *atpD* gene analyses (figures 2, 3 and 4).

In the genus *Ensifer*, also some species were indistinguishable, for example, *E. kummerowiae* CCBAU 71714^T^ matched with *E. meliloti* ATCC 9930^T^ with a score value of 2.261 suggesting that they belong to the same species (Table 2D). Since this result coincides with the analysis of *rrs*, *recA* and *atpD* genes, the taxonomic status of *E. kummerowiae* should be revised.

All these findings showed that MALDI-TOF MS results are comparable to those obtained after the phylogenetic analysis of core genes from members of family *Rhizobiaceae* including that of *rrs* gene in which is currently based the classification within this family [1]. These results are in agreement with those previously reported for other bacterial groups [37] and therefore we analysed the potential of MALDI-TOF MS for identification of fast-growing rhizobia isolates.

### Identification of rhizobial strains by MALDI-TOF MS

To prove the suitability of the extended MALDI Biotyper 2.0 database for routine identification and discrimination of fast-growing rhizobial species we analysed several strains previously identified by *rrs* and housekeeping gene sequencing belonging to different species and genera of family *Rhizobiaceae* (Table 2F and 2G).

The species *R. leguminosarum* contains some strains with identical *rrs* gene and divergent *recA* and *atpD* genes [29], [38], [39]. For example, the strains RPVF18, RVS11 and ATCC 14480 have housekeeping genes closely related to the type strain of this species USDA 2370^T^ and other strains have phylogenetically distant ones, such as RPVR31, CVIII4 and RTP05 (figures 3 and 4). Although all these strains clustered with *R. leguminosarum* USDA 2370^T^ after MALDI-TOF MS cluster analysis (figure 5), only when the housekeeping genes were almost identical the score values were higher than 2 with respect to *R. leguminosarum* USDA 2370^T^ (Table 2F). These results were congruent with those from *recA* and *atpD* gene analyses showing that, in spite of the complete identity of *rrs* gene, *R. leguminosarum* could contain several subspecies perfectly distinguishable by MALDI-TOF MS analysis as it has already been observed in other bacterial species [26], [40], [41].

Although housekeeping gene sequences present higher variability than those of *rrs* genes, the ITS fragment located between 16S and 23S gene in fast growing rhizobia is the most hypervariable chromosomic region and has been proposed as a tool for species differentiation [42]. However, MALDI-TOF MS showed that strains with housekeeping genes nearly identical but different ITS sequences belong to the same species. For example, the strains RPA12 and RPA02 shared only 73% identity in their ITS sequences with respect to *R. giardinii* H152^T^ suggesting they can represent different species [43]. However, in agreement with *rrs*, *recA* and *atpD* gene analyses, MALDI-TOF MS showed that they belong to *R. giardinii* since they matched with the type strain of this species with score values higher than 2 (Table 2F).

The same was found for the genus *Ensifer* strains RTM17 and GVPV12 that matched with *E. meliloti* ATCC 9930^T^ with score values higher than 2 (Table 2F) in spite of the differences in the ITS region (95% identity) [44] and in agreement with the results of the housekeeping gene analyses (figures 3 and 4).

Intraspecific variability in species of family *Rhizobiaceae* could be also due to the presence of large plasmids codifying for symbiotic or virulence factors. Nodulating species may contain different biovars that carry different *nodC* genes [21], [38], [44], [45] and pathogenic species contain strains that carry plasmids involved in tumour (pTi) or hairy roots induction (pRi) [46], [47]. Therefore we analysed strains with different combinations of chromosomal backgrounds and symbiotic or virulence plasmids by MALDI-TOF MS.

**Figure 7:**
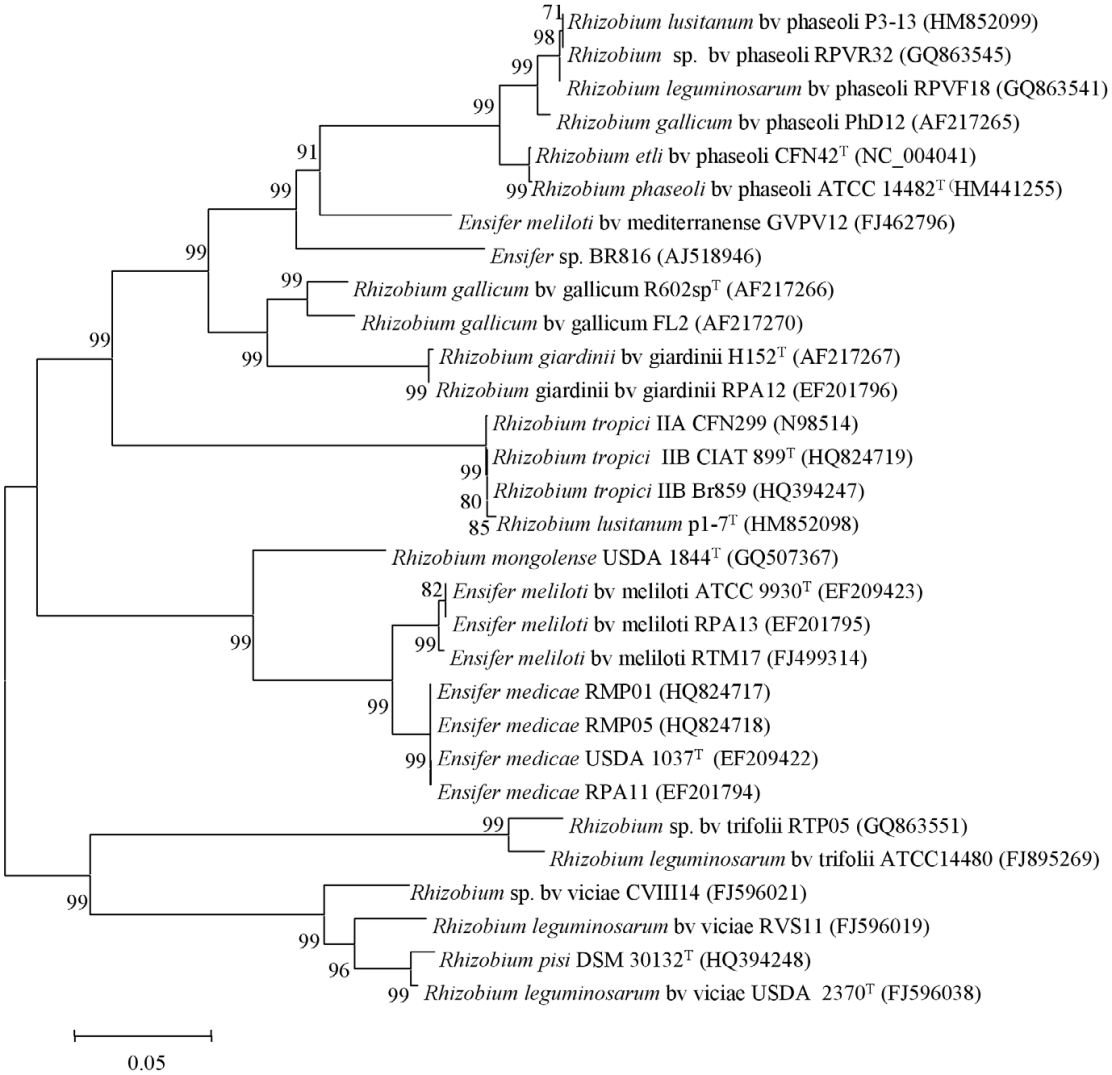
Neighbour-joining phylogenetic tree based on *nodC* gene sequences (about 860 nt) showing the position of representative strains from each biovar analysed in this study. Bootstrap values calculated for 1000 replications are indicated. Bar, 5 nt substitution per 100 nt. Accesion numbers from Genbank are given in brackets.

For example, within genus *Rhizobium*, *R. leguminosarum* contains three biovars: viciae, trifolii and phaseoli [31], [38], perfectly distinguishable on the basis of their *nodC* gene sequences (figure 7). However MALDI-TOF MS analysis showed that strains with housekeeping genes close to *R. leguminosarum* USDA 2370^T^ (RVS11, RPVF18 and ATCC 14480) [29], [38], [39] matched with score values higher than 2 with this strain with independence to the biovar they belong to (figure 7). Likewise, the strains FL27 from biovar gallicum [45] and PhD12 from biovar phaseoli [21] carrying divergent *nodC* genes (figure 7) matched with *R. gallicum* R602sp^T^ with score values higher than 2 (Table 2F). The same was found in *R. lusitanum* whose strains P1-7^T^ and P3-13 have phylogenetically distant *nodC* genes (figure 7) but they matched with a score value of 2.314 (Table 2F).

In genus *Ensifer*, *E. meliloti* also contains different biovars with divergent *nodC* genes (figure 7). However, the strains RPA13 and RTM17 from biovar meliloti and the strain GVPV12 from biovar mediterranense [44] were matched with *E. meliloti* ATCC 9930^T^ with score values higher than 2 by MALDI-TOF MS (Table 2F).

Conversely, strains from the same biovar but divergent housekeeping genes were perfectly distinguished by MALDI-TOF MS in genus *Rhizobium*. For example, the strain CVIII14 matched with a score value lower than 2 with *R. leguminosarum* USDA 2370^T^, although both strains belong to the biovar viciae (Table 2G). To this biovar also belongs *R. pisi* DSM 30132^T^ that was correctly distinguished by MALDI-TOF MS from *R. leguminosarum* USDA 2370^T^ (figure 7 and Table 2C). Moreover, strains CFN299 and CIAT 899^T^, whose *rrs* and housekeeping genes showed they belong to different species [48], matched with score values lower than 2 (Table 2F) in spite of the complete identity of their *nodC* genes (figure 7).

In genus *Ensifer*, *E. meliloti* RPA13 and RTM17 and *E. medicae* RMP01, RMP05, RPA08, RPA11 and RPA20 belong to the same biovar (meliloti) [49]. However, in agreement with *rrs* and housekeeping gene analyses (figures 2, 3 and 4), the strains of these both species were clearly distinguished by MALDI-TOF MS (Table 2F).

Finally, two species of genus *Rhizobium*, *R. rhizogenes* and *R. radiobacter*, contain non-pathogenic strains, tumourigenic strains and hairy roots inducing strains (Table 2F). In both cases their strains were correctly identified by MALDI-TOF MS in agreement with the *rrs* and housekeeping gene analyses (figures 2, 3 and 4) in spite of the plasmidic content. In this way the non-pathogenic strain K84 [46], the tumourigenic strain 163C and the root inducing strain IAM 13571, matched with *R. rhizogenes* ATCC 11325^T^, a root inducing strain, with high score values (2.185, 2.195 and 2.158, respectively). The tumourigenic strain ATCC 23308 (type strain of the former species *A. tumefaciens*) and the root inducing strain ATCC 13332 (erroneously named *R. rhizogenes*) also matched with the non-pathogenic strain *R. radiobacter* DSM 30147^T^ with score values higher than 2 (Table 2F).

Conversely, although the pTi plasmids of the tumourigenic strains 163C and C58 are closely related [47], they belong to different species according to MALDI-TOF MS results (Table 2G) in agreement with the *rrs* and housekeeping gene analyses (figures 2, 3 and 4).

All these results showed that plasmids carried by fast growing rhizobial strains do not affect their identification by MALDI-TOF MS since strains of the same species carrying very different plasmids and strains from different species carrying similar plasmids were correctly identified by MALDI-TOF MS.

In conclusion, the results presented in this work clearly showed that MALDI-TOF MS is a reliable and rapid method for rhizobial identification comparable to housekeeping gene sequence analysis since it is able to discriminate between strains with identical *rrs* gene sequences but divergent *recA* and *atpD*. This feature represents important advantages based on the rapidity and cost per sample with respect to gene sequencing. With this methodology, if the databases include all rhizobial species described in each moment, it will be possible to identify all isolates belonging to species already described as well as the detection of new species. Therefore, MALDI-TOF MS open a new and very useful way for diversity and ecological studies applicable to analysis of large populations of isolates allowing the differentiation of strains, species and genera of fast-growing rhizobia with an effectiveness of 100% in the identification at species level.

## References

[1] Kuykendall LD, Brenner DJ, Krieg NR, Stanley JT (2005). Family I. Rhizobiaceae Conn 1938, 321^AL^.. Bergey's Manual of Systematic Bacteriology. Vol. 2, part C.

[2] Lin DX, Wang ET, Tang H, Han TX, He YR (2008). *Shinella kummerowiae* sp. nov., a symbiotic bacterium isolated from root nodules of the herbal legume *Kummerowia stipulacea*.. Int J Syst Evol Microbiol.

[3] Young JM, Kuykendall LD, Martínez-Romero E, Kerr A, Sawada H (2001). A revision of *Rhizobium* Frank 1889, with an emended description of the genus, and the inclusion of all species of *Agrobacterium* Conn 1942 and *Allorhizobium undicola* de Lajudie et al. 1998 as new combinations: *Rhizobium radiobacter*, *R. rhizogenes*, *R. rubi*, *R. undicola* and *R. vitis*.. Int J Syst Evol Microbiol.

[4] Judicial Commission of the International Committee on Systematics of Prokaryotes (2008). The genus name *Sinorhizobium* Chen et al. 1988 is a later synonym of *Ensifer* Casida 1982 and is not conserved over the latter genus name, and the species name *‘Sinorhizobium adhaerens’* is not validly published. Opinion 84.. Int J Syst Evol Microbiol.

[5] Seng P, Drancourt M, Gouriet F, La Scola B, Fournier PE (2009). Ongoing revolution in bacteriology: routine identification of bacteria by matrix-assisted laser desorption ionization time-of-flight mass spectrometry.. Clin Infect Dis.

[6] Bizzini A, Durussel C, Bille J, Greub G, Prod'hom G (2010). Performance of matrix-assisted laser desorption/ionization time-of-flight mass spectrometry for the identification of bacterial strains routinely isolated in a clinical microbiology laboratory.. J Clin Microbiol.

[7] Cherkaoui A, Hibbs J, Emonet S, Tangomo M, Girard M (2010). Comparison of two matrix-assisted laser desorption ionization-time of flight mass spectrometry methods with conventional phenotypic identification for routine identification of bacteria to the species level.. J Clin Microbiol.

[8] Giebel R, Worden C, Rust SM, Kleinheinz GT, Robbins M (2010). Microbial fingerprinting using matrix-assisted laser desorption ionization time-of-flight mass spectrometry (MALDI-TOF MS) Applications and Challenges.. Adv Appl Microbiol.

[9] Ferreira L, Sánchez-Juanes F, González-Avila M, Cembrero-Fuciños D, Herrero-Hernández A (2010). Direct identification of urinary tract pathogens from urine samples by matrix-assisted laser desorption ionization-time of flight mass spectrometry.. J Clin Microbiol.

[10] Ferreira L, Vega S, Sánchez-Juanes F, González M, Herrero A (2010). Identifying bacteria using a matrix-assisted laser desorption ionization time-of-flight (MALDI-TOF) mass spectrometer. Comparison with routine methods used in clinical microbiology laboratories.. Enferm Infecc Microbiol Clin.

[11] Ferreira L, Sánchez-Juanes F, Guerra IP, García García MI, Sánchez JE (2011). Microorganisms direct identification from blood culture by maldi-tof mass spectrometry.. Clin Microbiol Infect.

[12] Ferreira L, Sánchez-Juanes F, Muñoz-Bellido JL, González-Buitrago JM (2010). Rapid method for direct identification of bacteria in urine and blood culture samples by MALDI-TOF MS: intact cell vs. extraction method.. Clin Microbiol Infect.

[13] Ferreira L, Vega-Castaño S, Sánchez-Juanes F, González-Cabrero S, Menegotto F (2010). Identification of *Brucella* by MALDI-TOF Mass Spectrometry. Fast and Reliable Identification from Agar Plates and Blood Cultures.. PLoS ONE.

[14] Mellmann A, Cloud J, Maier T, Keckevoet U, Ramminger I (2008). Evaluation of matrix-assisted laser desorption ionization-time-of-flight mass spectrometry in comparison to 16S rRNA gene sequencing for species identification of nonfermenting bacteria.. J Clin Microbiol.

[15] Stevenson LG, Drake SK, Murray PR (2010). Rapid identification of bacteria in positive blood culture broths by matrix-assisted laser desorption ionization-time of flight mass spectrometry.. J Clin Microbiol.

[16] van Veen SQ, Claas EC, Kuijper EJ (2010). High-throughput identification of bacteria and yeast by matrix-assisted laser desorption ionization-time of flight mass spectrometry in conventional medical microbiology laboratories.. J Clin Microbiol.

[17] Beringer JE (1974). R factors transfer in *Rhizobium leguminosarum*.. J Gen Microbiol.

[18] Vincent JM, Vincent JM (1970). The cultivation, isolation and maintenance of rhizobia.. A Manual for the Practical Study of Root-Nodule.

[19] Rivas R, García-Fraile P, Mateos PF, Martínez-Molina E, Velázquez E (2007). Characterization of xylanolytic bacteria present in the bract phyllosphere of the date palm *Phoenix dactylifera*.. Lett Appl Microbiol.

[20] Gaunt MW, Turner SL, Rigottier-Gois L, Lloyd-Macgilp SA, Young JWP (2001). Phylogenies of *atpD* and *recA* support the small subunit rRNA-based classification of rhizobia.. Int J Syst Evol Microbiol.

[21] Laguerre G, Nour SM, Macheret V, Sanjuan J, Drouin P (2001). Classification of rhizobia based on *nodC* and *nifH* gene analysis reveals a close phylogenetic relationship among *Phaseolus vulgaris* symbionts.. Microbiology.

[22] Thompson JD, Gibson TJ, Plewniak F, Jeanmougin F, Higgins DG (1997). The clustalX windows interface: flexible strategies for multiple sequence alignement aided by quality analysis tools.. Nucleic Acid Res.

[23] Kimura M (1980). A simple method for estimating evolutionary rates of base substitutions through comparative studies of nucleotide sequences.. J Mol Evol.

[24] Saitou N, Nei M (1987). A neighbour-joining method: a new method for reconstructing phylogenetics trees.. Mol Biol Evol.

[25] Tamura K, Dudley J, Nei M, Kumar S (2007). MEGA4: Molecular Evolutionary Genetics Analysis (MEGA) software version 4.0.. Mol Biol Evol.

[26] Seibold E, Maier T, Kostrzewa M, Zeman E, Splettstoesser W (2010). Identification of *Francisella tularensis* by whole-cell matrix-assisted laser desorption ionization-time of flight mass spectrometry: fast, reliable, robust, and cost-effective differentiation on species and subspecies levels.. J Clin Microbiol.

[27] Barbuddhe SB, Maier T, Schwarz G, Kostrzewa M, Hof H (2008). Rapid identification and typing of listeria species by matrix-assisted laser desorption ionization-time of flight mass spectrometry.. Appl Environ Microbiol.

[28] Sawada H, Ieki H, Oyaizu H, Matsumoto S (1993). Proposal for rejection of *Agrobacterium tumefaciens* and revised descriptions for the genus *Agrobacterium* and for *Agrobacterium radiobacter* and *Agrobacterium rhizogenes*.. Int J Syst Bacteriol.

[29] Ramírez-Bahena MH, García-Fraile P, Peix A, Valverde A, Rivas R (2008). Revision of the taxonomic status of the species *Rhizobium leguminosarum* (Frank 1879) Frank 1889, *R. phaseoli* Dangeard 1926^AL^ and *R. trifolii* Dangeard 1926^AL^. *R. trifolii* is a later synonym of *R. leguminosarum*. Reclassification of the strain *Rhizobium leguminosarum* DSM 30132^T^ ( = NCIMB 11478^T^) into the new species *Rhizobium pisi* sp. nov.. Int J Syst Evol Microbiol.

[30] Kaur J, Verma M, Lal R (2010). *Rhizobium rosettiformans* sp. nov., isolated from hexachlorocyclohexane (HCH) dump site in India, and reclassification of *Blastobacter aggregatus* Hirsch and Müller (1985) as *Rhizobium aggregatum* comb. nov.. Int J Syst Evol Microbiol.

[31] Jordan DC, Krieg NR, Holt JG (1984). Family III. *Rhizobiaceae*.. Bergey's Manual of Systematic Bacteriology.

[32] Segovia L, Young JP, Martínez-Romero E (1993). Reclassification of American *Rhizobium leguminosarum* biovar phaseoli type I strains as *Rhizobium etli* sp. nov.. Int J Syst Bacteriol.

[33] Martens M, Dawyndt P, Coopman R, Gillis M, de Vos P (2008). Advantages of multilocus sequence analysis for taxonomic studies: a case study using 10 housekeeping genes in the genus *Ensifer* (including former *Sinorhizobium*).. Int J Syst Evol Microbiol.

[34] van Rhijn PJ, Feys B, Verreth C, Vanderleyden J (1993). Multiple copies of *nodD* in *Rhizobium tropici* CIAT899 and BR816.. J Bacteriol.

[35] Snoeck C, Verreth C, Hernández-Lucas I, Martínez-Romero E, Vanderleyden J (2003). Identification of a third sulfate activation system in *Sinorhizobium* sp. strain BR816: the CysDN sulfate activation complex.. Appl Environ Microbiol.

[36] Young JM (2003). The genus name *Ensifer* Casida 1982 takes priority over *Sinorhizobium* Chen et al. 1988, and *Sinorhizobium morelense* Wang et al. 2002 is a later synonym of *Ensifer adhaerens* Casida 1982. Is the combination “*Sinorhizobium adhaerens*” Casida 1982 Willems et al. 2003 legitimate? Request for an Opinion.. Int J Syst Evol Microbiol.

[37] Murray PR (2010). Matrix-assisted laser desorption ionization time-of-flight mass spectrometry: usefulness for taxonomy and epidemiology.. Clin Microbiol Infect.

[38] García-Fraile P, Mulas-García D, Peix A, Rivas R, González-Andrés F (2010). *Phaseolus vulgaris* is nodulated in northern Spain by *Rhizobium leguminosarum* strains harboring two *nodC* alleles present in American *Rhizobium etli* strains: biogeographical and evolutionary implications.. Can J Microbiol.

[39] Alvarez-Martínez ER, Valverde A, Ramírez-Bahena MH, García-Fraile P, Tejedor C (2009). The analysis of core and symbiotic genes of rhizobia nodulating *Vicia* from different continents reveals their common phylogenetic origin and suggests the distribution of *Rhizobium leguminosarum* strains together with *Vicia* seeds.. Arch Microbiol.

[40] Dieckmann R, Helmuth R, Erhard M, Malorny B (2008). Rapid classification and identification of Salmonellae at the species and subspecies levels by whole cell matrix-assisted laser desorption ionization time of flight mass spectrometry.. Appl Environ Microbiol.

[41] Tanigawa K, Kawabata H, Watanabe K (2010). Identification and typing of *Lactococcus lactis* by matrix-assisted laser desorption ionization-time of-flight mass spectrometry.. Appl Environ Microbiol.

[42] Kwon SW, Park JY, Kim JS, Kang JW, Cho YH (2005). Phylogenetic analysis of the genera *Bradyrhizobium*, *Mesorhizobium*, *Rhizobium* and *Sinorhizobium* on the basis of 16S rRNA gene and internally transcribed spacer region sequences.. Int J Syst Evol Microbiol.

[43] Iglesias O, Rivas R, García-Fraile P, Abril A, Mateos PF (2007). Genetic characterization of fast-growing rhizobia able to nodulate *Prosopis alba* in North Spain.. FEMS Microbiol Lett.

[44] Zurdo-Piñeiro JL, García-Fraile P, Rivas R, Peix A, León-Barrios M (2009). Rhizobia from Lanzarote, the Canary Islands, that nodulate *Phaseolus vulgaris* have characteristics in common with *Sinorhizobium meliloti* isolates from mainland Spain.. Appl Environ Microbiol.

[45] Amarger N, Macheret V, Laguerre G (1997). *Rhizobium gallicum* sp. nov. and *Rhizobium giardinii sp*. nov., from *Phaseolus vulgaris* nodules.. Int J Syst Bacteriol.

[46] Velázquez E, Palomo JL, Rivas R, Guerra H, Peix A (2010). Analysis of core genes supports the reclassification of strains *Agrobacterium radiobacter* K84 and *Agrobacterium tumefaciens* AKE10 into the species *Rhizobium rhizogenes*.. Syst Appl Microbiol.

[47] Velázquez E, Peix A, Zurdo-Piñeiro JL, Palomo JL, Mateos PF (2005). The coexistence of symbiosis and pathogenicity-determining genes in *Rhizobium rhizogenes* strains enables them to induce nodules and tumors or hairy roots in plants.. Mol Plant Microbe Interact.

[48] Valverde A, Igual JM, Peix A, Cervantes E, Velázquez E (2006). *Rhizobium lusitanum* sp. nov. a bacterium that nodulates *Phaseolus vulgaris*.. Int J Syst Evol Microbiol.

[49] del Villar M, Rivas R, Peix A, Mateos PF, Martínez-Molina E (2008). Stable low molecular weight RNA profiling showed variations within *Sinorhizobium meliloti* and *Sinorhizobium medicae* nodulating different legumes from the alfalfa cross-inoculation group.. FEMS Microbiol Lett.

[50] Casida LEJ (1982). *Ensifer adhaerens* gen nov. sp. nov.: a bacterial predator of bacteria in soil.. Int J Syst Bacteriol.

[51] Toledo I, Lloret L, Martínez-Romero E (2003). *Sinorhizobium americanus* sp. nov., a new *Sinorhizobium* species nodulating native *Acacia* spp. in Mexico.. Syst Appl Microbiol.

[52] Nick G, de Lajudie P, Eardly BD, Suomalainen S, Paulin L (1999). *Sinorhizobium arboris* sp. nov. and *Sinorhizobium kostiense* sp. nov., isolated from leguminous trees in Sudan and Kenya.. Int J Syst Bacteriol.

[53] Scholla MH, Elkan GH (1984). *Rhizobium fredii* sp. nov., a Fast-Growing Species That Effectively Nodulates Soybeans.. Int J Syst Bacteriol.

[54] Chen WX, Yan GH, Li JL (1988). Numerical taxonomy study of fast-growing soybean rhizobia and a proposal that *Rhizobium fredii* be assigned to *Sinorhizobium* gen. nov.. Int J Syst Bacteriol.

[55] Merabet C, Martens M, Mahdhi M, Zakhia F, Sy A (2010). Multilocus sequence analysis of root nodule isolates from *Lotus arabicus* (Senegal), *Lotus creticus*, *Argyrolobium uniflorum* and *Medicago sativa* (Tunisia) and description of *Ensifer numidicus* sp. nov. and *Ensifer garamanticus* sp. nov.. Int J Syst Evol Microbiol.

[56] Wei GH, Wang ET, Tan ZY, Zhu ME, Chen WX (2002). *Rhizobium indigoferae* sp. nov. and *Sinorhizobium kummerowiae* sp. nov., respectively isolated from *Indigofera* spp. and *Kummerowia stipulacea*.. Int J Syst Evol Microbiol.

[57] Rome S, Fernandez MP, Brunel B, Normand P, Cleyet-Marel JC (1996). *Sinorhizobium medicae* sp. nov., isolated from annual *Medicago* spp.. Int J Syst Bacteriol.

[58] de Lajudie P, Willems A, Pot B, Dewettinck D, Maestrojuan G (1994). Polyphasic taxonomy of Rhizobia: emendation of the genus *Sinorhizobium* and description of *Sinorhizobium meliloti* comb. nov., *Sinorhizobium saheli* sp. nov., and *Sinorhizobium teranga* sp. nov.. Int J Syst Bacteriol.

[59] Wang ET, Tan ZY, Willems A, Fernández-López M, Reinhold-Hurek B (2002). *Sinorhizobium morelense* sp. nov., a *Leucaena leucocephala*-associated bacterium that is highly resistant to multiple antibiotics.. Int J Syst Evol Microbiol.

[60] Hirsch P, Müller M (1985). Title *Blastobacter aggregatus* sp. nov., *Blastobacter capsulatus* sp. nov., and *Blastobacter denitrificans* sp. nov., new budding bacteria from freshwater habitats.. Syst Appl Microbiol.

[61] Berge O, Lodhi A, Brandelet G, Santaella C, Roncato MA (2009). *Rhizobium alamii* sp. nov., an exopolysaccharide-producing species isolated from legume and non-legume rhizospheres.. Int J Syst Evol Microbiol.

[62] Lu YL, Chen WF, Han LL, Wang ET, Chen WX (2009). *Rhizobium alkalisoli* sp. nov., isolated from *Caragana intermedia* growing in saline-alkaline soils in the north of China.. Int J Syst Evol Microbiol.

[63] Zhang GX, Ren SZ, Xu MY, Zeng GQ, Luo HD (2010). *Rhizobium borbori* sp. nov., an aniline-degrading bacterium isolated from activated sludge.. Int J Syst Evol Microbiol.

[64] García-Fraile P, Rivas R, Willems A, Peix A, Martens M (2007). *Rhizobium cellulosilyticum* sp. nov., isolated from sawdust of *Populus alba*.. Int J Syst Evol Microbiol.

[65] Quan ZX, Bae HS, Baek JH, Chen WF, Im WT (2005). *Rhizobium daejeonense* sp. nov. isolated from a cyanide treatment bioreactor.. Int J Syst Evol Microbiol.

[66] Tian CF, Wang ET, Wu LJ, Han TX, Chen WF (2009). *Rhizobium fabae* sp. nov., a bacterium that nodulates *Vicia faba*.. Int J Syst Evol Microbiol.

[67] Lindström K (1989). *Rhizobium galegae*, a new species of legume root nodule bacteria.. Int J Syst Bacteriol.

[68] Chen WX, Tan ZY, Gao JL, Li Y, Wang ET (1997). *Rhizobium hainanense* sp. nov., isolated from tropical legumes.. Int J Syst Bacteriol.

[69] Wang ET, van Berkum P, Beyene D, Sui XH, Dorado O (1998). *Rhizobium huautlense* sp. nov., a symbiont of *Sesbania herbacea* that has a close phylogenetic relationship with *Rhizobium galegae*.. Int J Syst Bacteriol.

[70] Bouzar H, Jones JB (2001). *Agrobacterium larrymoorei* sp. nov., a pathogen isolated from aerial tumours of *Ficus benjamina*.. Int J Syst Evol Microbiol.

[71] Young JM (2004). Renaming of *Agrobacterium larrymoorei* Bouzar and Jones 2001 as *Rhizobium larrymoorei* (Bouzar and Jones 2001) comb. nov.. Int J Syst Evol Microbiol.

[72] Wei GH, Tan ZY, Zhu ME, Wang ET, Han SZ (2003). Characterization of rhizobia isolated from legume species within the genera *Astragalus* and *Lespedeza* grown in the Loess Plateau of China and description of *Rhizobium loessense* sp. nov.. Int J Syst Evol Microbiol.

[73] Lin DX, Chen WF, Wang FQ, Hu D, Wang ET (2009). *Rhizobium mesosinicum* sp. nov., isolated from root nodules of three different legumes.. Int J Syst Evol Microbiol.

[74] Gu CT, Wang ET, Tian CF, Han TX, Chen WF (2008). *Rhizobium miluonense* sp. nov., a symbiotic bacterium isolated from *Lespedeza* root nodules.. Int J Syst Evol Microbiol.

[75] van Berkum P, Beyene D, Bao G, Campbell TA, Eardly BD (1998). *Rhizobium mongolense* sp. nov. is one of three rhizobial genotypes identified which nodulate and form nitrogen-fixing symbioses with *Medicago ruthenica* [(L.) Ledebour].. Int J Syst Bacteriol.

[76] Han TX, Wang ET, Wu LJ, Chen WF, Gu JG (2008). *Rhizobium multihospitium* sp. nov., isolated from multiple legume species native of Xinjiang, China.. Int J Syst Evol Microbiol.

[77] Peng G, Yuan Q, Li H, Zhang W, Tan Z (2008). *Rhizobium oryzae* sp. nov., isolated from the wild rice *Oryza alta*.. Int J Syst Evol Microbiol.

[78] Hildebrand EM (1940). Cane gall of brambles caused by *Phytomonas* n. sp.. J Agric Res.

[79] Hunter WJ, Kuykendall LD, Manter DK (2007). *Rhizobium selenireducens* sp. nov.: A Selenite-Reducing alpha-Proteobacteria Isolated From a Bioreactor.. Curr Microbiol.

[80] Squartini A, Struffi P, Döring H, Selenska-Pobell S, Tola E (2002). *Rhizobium sullae* sp. nov. (formerly *‘Rhizobium hedysari’*), the root-nodule microsymbiont of *Hedysarum coronarium* L.. Int J Syst Evol Microbiol.

[81] Hou BC, Wang ET, Li Y, Jia RZ, Chen WF (2009). *Rhizobium tibeticum* sp. nov., a symbiotic bacterium isolated from *Trigonella archiducis-nicolai* (Sirj.) Vassilcz.. Int J Syst Evol Microbiol.

[82] Martínez-Romero E, Segovia L, Mercante FM, Franco AA, Graham P (1991). *Rhizobium tropici*, a novel species nodulating *Phaseolus vulgaris* L. beans and *Leucaena* sp. trees.. Int J Syst Bacteriol.

[83] Zhang RJ, Hou BC, Wang ET, Li Y, Zhang XX (2010). *Rhizobium tubonense* sp. nov., a symbiotic bacterium isolated from root nodules of *Oxytropis glabra* grown in Tibet, China.. Int J Syst Evol Microbiol.

[84] de Lajudie P, Laurent-Fulele E, Willems A, Torck U, Coopman R (1992). *Allorhizobium undicola* gen. nov., sp. nov., nitrogen-fixing bacteria that efficiently nodulate *Neptunia natans* in Senegal.. Int J Syst Bacteriol.

[85] Ophel K, Kerr A (1990). *Agrobacterium vitis* sp. nov. for Strains of Agrobacterium biovar 3 from Grapevines.. Int J Syst Bacteriol.

[86] Ren DW, Chen WF, Sui XH, Wang ET, Chen WX (2010). *Rhizobium vignae* sp. nov., a symbiotic bacterium isolated from multiple legume species grown in China.. Int J Syst Evol Microbiol.

[87] Tan ZY, Kan F, Peng GX, Wang ET, Reinhold-Hurek B (2001). *Rhizobium yanglingense* sp. nov., isolated from arid and semi-arid regions in China.. Int J Syst Evol Microbiol.

[88] An DS, Im WT, Yang HC, Lee ST (2006). *Shinella granuli* gen. nov., sp. nov., and proposal of the reclassification of *Zoogloea ramigera* ATCC 19623 as *Shinella zoogloeoides* sp. nov.. Int J Syst Evol Microbiol.

[89] Vaz-Moreira I, Faria C, Lopes AR, Svensson LA, Moore ERB (2010). *Shinella fusca* sp. nov., isolated from domestic waste compost.. Int J Syst Evol Microbiol.

[90] Matsui T, Shinzato N, Tamaki H, Muramatsu M, Hanada S (2009). *Shinella yambaruensis* sp. nov., a 3-methyl-sulfolane-assimilating bacterium isolated from soil.. Int J Syst Evol Microbiol.

[91] Crabtree K, McCoy E (1967). *Zoogloea ramigera* Itzigsohn, identification and description.. Int J Syst Bacteriol.

[92] Peng GX, Tan ZY, Wang ET, Reinhold-Hurek B, Chen WF (2002). Identification of isolates from soybean nodules in Xinjiang Region as *Sinorhizobium xinjiangense* and genetic differentiation of *S. xinjiangense* from *Sinorhizobium fredii*.. Int J Syst Evol Microbiol.

[93] Velázquez E, Palomo JL, Lastra B, Mateos P, García-Benavides P (2001). Rapid identification of *Agrobacterium* species by staircase electrophoresis of low molecular weight RNA profiles.. Eur J Plant Pathol.

[94] Ramírez-Bahena MH, Velázquez E, Fernández-Santos F, Peix A, Martínez-Molina E (2009). Phenotypic, genotypic and symbiotic diversity in strains nodulating clover in different soils in Spain.. Can J Microbiol.

[95] Goodner B, Hinkle G, Gattung S, Miller N, Blanchard M (2001). Genome sequence of the plant pathogen and biotechnology agent *Agrobacterium tumefaciens* C58.. Science.

